# Extraction and Biological Activity of Lignanoids from *Magnolia officinalis* Rehder & E.H.Wilson Residual Waste Biomass Using Deep Eutectic Solvents

**DOI:** 10.3390/molecules29102352

**Published:** 2024-05-16

**Authors:** Ying Lu, Haishan Tang, Feng Chen, Wufei Tang, Wubliker Dessie, Yunhui Liao, Zuodong Qin

**Affiliations:** 1 College of Chemistry and Bioengineering, Hunan University of Science and Engineering, Yongzhou 425199, China; 2 Hunan Engineering Technology Research Center for Comprehensive Development and Utilization of Biomass Resources, Yongzhou 425199, China; 3 Hunan Provincial Key Laboratory for Comprehensive Utilization of Dominant Plant Resources in Southern Hunan, Yongzhou 425199, China

**Keywords:** deep eutectic solvents, natural product extraction, magnolol, honokiol, biological activity

## Abstract

Lignanoids are an active ingredient exerting powerful antioxidant and anti-inflammatory effects in the treatment of many diseases. In order to improve the efficiency of the resource utilization of traditional Chinese medicine waste, *Magnolia officinalis* Rehder & E.H.Wilson residue (MOR) waste biomass was used as raw material in this study, and a series of deep eutectic solvents (ChUre, ChAce, ChPro, ChCit, ChOxa, ChMal, ChLac, ChLev, ChGly and ChEG) were selected to evaluate the extraction efficiency of lignanoids from MORs. The results showed that the best conditions for lignanoid extraction were a liquid–solid ratio of 40.50 mL/g, an HBD-HBA ratio of 2.06, a water percentage of 29.3%, an extract temperature of 337.65 K, and a time of 107 min. Under these conditions, the maximum lignanoid amount was 39.18 mg/g. In addition, the kinetics of the extraction process were investigated by mathematic modeling. In our antioxidant activity study, high antioxidant activity of the lignanoid extract was shown in scavenging four different types of free radicals (DPPH, ·OH, ABTS, and superoxide anions). At a concentration of 3 mg/mL, the total antioxidant capacity of the lignanoid extract was 1.795 U/mL, which was equal to 0.12 mg/mL of V_c_ solution. Furthermore, the antibacterial activity study found that the lignanoid extract exhibited good antibacterial effects against six tested pathogens. Among them, *Staphylococcus aureus* exerted the strongest antibacterial activity. Eventually, the correlation of the lignanoid extract with the biological activity and physicochemical properties of DESs is described using a heatmap, along with the evaluation of the in vitro hypoglycemic, in vitro hypolipidemic, immunomodulatory, and anti-inflammatory activity of the lignanoid extract. These findings can provide a theoretical foundation for the extraction of high-value components from waste biomass by deep eutectic solvents, as well as highlighting its specific significance in natural product development and utilization.

## 1. Introduction

**Figure 1:**
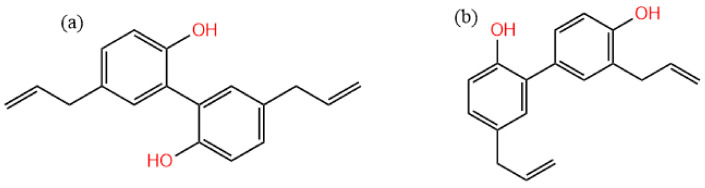
The molecular structure of (**a**) magnolol and (**b**) honokiol.

In recent years, the number of medicinal residues has increased dramatically with the rapid development of large health industries, which greatly rely on traditional Chinese medicine and natural medicine resources as raw materials [1]. However, these residues and other wastes have not been effectively utilized due to the constraints of extraction technology, equipment, and management factors [1,2]. Usually, the extraction solvent of traditional Chinese medicine is an aqueous solution, and there is still a large number of fat-soluble components remaining in the residues. *Magnolia* bark is one of the three Chinese wood medicinal materials (*Magnolia officinalis*, *Eucommia ulmoides*, and *Phellodendron amurense*) with main clinical pharmacological effects such as antibacterial [3,4], anti-inflammatory [5,6], anti-tumor [7,8], neuroprotective [9,10], blood-quickening, and stasis-dispelling effects [11]. The main components of *Magnolia officinalis* are lignanoids, including honokiol, isomagnolol, trihydroxy magnolol, and dehydrotrihydroxy magnolol [12,13]. Among them, magnolol (Figure 1a) and honokiol (Figure 1b) are the active ingredients with the highest content in *Magnolia officinalis*, and they are standard detection substances for evaluating the quality of *Magnolia officinalis* [14,15]. There are several potential application prospects in the extraction of lignanoids from *Magnolia officinalis*, which have become a hot spot in the research of biomass resources and the environment industry [16,17]. The extraction process of traditional Chinese medicine components is mainly based on decoction, which can dissolve some water-soluble functional components, and there are still more active components in *Magnolia officinalis* residues (MORs) [18]. As a typical functional ingredient of *Magnolia officinalis*, the extraction and utilization of lignanoids has attracted much attention [12,19]. The main extraction methods include alcohol extraction [20,21], water extraction [22,23], alkali-dissolved acid precipitation [24,25], etc. These methods are simple to operate and do not require complicated equipment, and are currently the main methods used in industry. However, there are some problems with the large amount of solvent used, long extraction time, and high extraction temperature [26,27]. Therefore, it is of great significance to find an efficient, green, and easy-to-prepare extraction solvent for the extraction of residual functional components in traditional Chinese medicine residues [28,29].

A deep eutectic solvent (DES) is a eutectic mixture formed of two or more components forming hydrogen bonds [30,31]; the melting point is usually lower than that of its constituent pure substance [32]. Since it is liquid at room temperature and has similar functions to traditional solvents, it has attracted widespread attention. The components of DESs include a hydrogen bond donor (HBD) and hydrogen bond acceptor (HBA), which can be biodegraded and meet the requirements of green chemistry [33,34], and different HBD-HBA ratios make an important contribution to the properties of DESs [35,36]. Moreover, DESs have a simple preparation process and strong designability, and are expected to become a green substitute for traditional solvents [37], although there have been some eutrophication problems [38]. Compared with conventional solvents, DESs have a stronger penetration ability, and the dissolution rate of active ingredients can be increased by assisting in the decomposition of plant cell wall lignin, hemicellulose, and cellulose [39,40].

This experiment takes *Magnolia officinalis* residues (MORs) as the extraction research object; the extraction efficiency of various DESs on residual lignanoids from MORs was investigated, and the effects of the main influencing factors on magnolol and honokiol were studied. The extraction procedure was optimized to enhance the extraction effect of lignanoids, offer a theoretical basis for the efficient utilization of MORs, and enlarge the application fields of DESs. Lastly, the biological activity of lignanoid extracts was systematically evaluated. These results offer a theoretical foundation for the utilization of natural products.

## 2. Results and Discussion

### 2.1. Optimal DES System for Lignanoid Extraction

The dissolution of active substances is significantly influenced by the composition of DESs, and the physical and chemical properties of mixtures prepared with different components vary greatly. Therefore, the lignanoid extraction yield of 10 DESs with different acids, alcohols, and ketones as HBDs from *Magnolia officinalis* residues was investigated. Following extraction, it was discovered that the amount of lignanoids increased simultaneously with the growth of the specific surface area (S_BET_) and pore volume (V_pore_), as shown in Table 1. There was damage to the cell wall structure, which made it easier for the DES to penetrate into the cells, thereby decreasing the barrier to mass transfer and increasing lignanoid leaching. The highest total extraction rate of lignanoids from *Magnolia officinalis* residues by choline chloride/levulinic acid (ChLev) was 28.35 mg/g, which presumably related to the ability of ChLev to destruct the structure of the cell wall. The intermolecular interactions between solutes and solvents might be influenced by the pH of DESs, leading to the different extraction efficiency from the sample matrix [41]. Thus, as shown in Table 1, the extraction yields of lignanoids were lower in super-acidic or neutral DESs, while the Chlev showed superior extraction efficiency than other DESs at a pH of about 1.

The extraction efficiency of choline chloride/propionic acid (ChPro) and choline chloride/acetic acid (ChAce) for lignanoids was only inferior to that of the ChLev solvent, with total extraction yields of 24.57 and 20.93 mg/g, respectively. The extraction efficiency of the remaining DESs using choline chloride as an HBA for lignanoids was relatively low. No magnolol components were detected in the extract of choline chloride/urea (ChUre). Therefore, only the extraction rates of the corresponding extracted substances were calculated. Based on the test results, ChLev was chosen as the next study object.

### 2.2. Single-Factor Investigation of the Extraction Process

**Figure 2:**
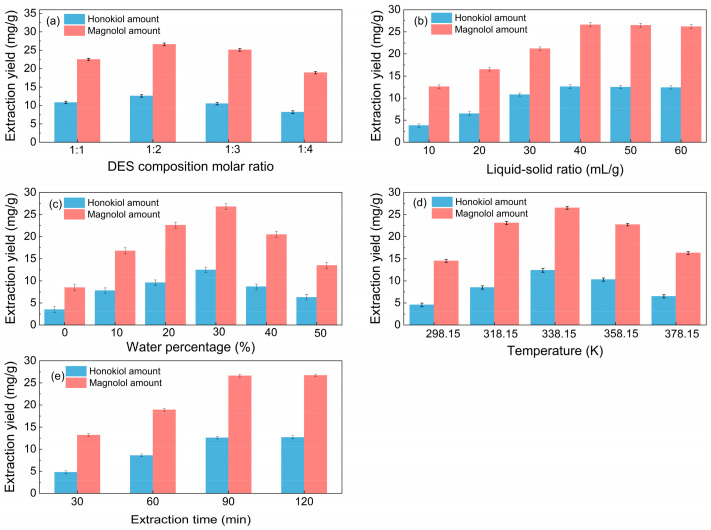
Single-factor investigation of the extraction process (**a**): liquid–solid ratio of 40 mL/g, water percentage of 30%, temperature of 338.15 K, and extraction time of 90 min; (**b**): DES composition molar ratio of 1:2, water percentage of 30%, temperature of 338.15 K, and extraction time of 90 min; (**c**): DES composition molar ratio of 1:2, liquid–solid ratio of 40 mL/g, temperature of 338.15 K, and extraction time of 90 min; (**d**): DES composition molar ratio of 1:2, liquid–solid ratio of 40 mL/g, water percentage of 30%, and extraction time of 90 min. (**e**): DES composition molar ratio of 1:2, liquid–solid ratio of 40 mL/g, water percentage of 30%, and temperature of 338.15 K.

Different molar ratios of choline chloride to hydrogen bond donors can affect the surface tension and viscosity of solvents, hence influencing the extraction efficiency. Thus, the impact of the DES composition molar ratio on the extraction performance was investigated; Figure 2a shows that the optimal DES composition (HBA-HBD) molar ratio is 1:2.

From Figure 2b, it is noticeable that when the liquid–solid ratio increased, the amount of total lignanoids initially increased and subsequently stabilized. It can be seen from Figure 2b that the yield of total lignanoids shows a trend of first increasing and then stabilizing with the increase in the liquid–solid ratio, and an inflection point appeared at the liquid–solid ratio of 40 mL/g. A suitable liquid–solid ratio makes it easier to extract chemical components. The total lignanoids were extracted completely once the liquid–solid ratio was 40 mL/g, so the optimum liquid–solid ratio is 40 mL/g.

The impact of water percentage on lignanoid extraction is depicted in Figure 2c. The lignanoid concentrations increased with the increased water volume fraction, which reached the highest level at the percentage of 30%. The reason may be that the high water content increased the polarity of the extraction medium, which destroyed the hydrogen bond between the DES and lignanoids, thus causing a negative impact on the extraction of lignanoids [42,43].

Extract temperature is critical for efficient mass transfer. Figure 2d shows that the lignanoid amount increases up to 338.15 K and then decreases from 358.15 K onwards. One probable explanation for this might be that when temperature rose, the thermal impact strengthened and sped up the dissolution of lignanoids extracted from the cells. However, as the temperature further increased, some lignanoids were destroyed and the dissolution of impurities increased, thereby reducing the extraction rate of lignanoids.

As shown in Figure 2e, the extraction amount of total lignanoids initially increased and subsequently drops with the increase in the extraction time. The reason may be that the extension of the extraction time helped to improve the concentrations of the solvent and solute. However, with the prolongation of time, some of the lignanoids were decomposed, so the extraction rate decreased. Therefore, the optimal extract time is selected as 90 min.

### 2.3. Response Surface Methodology (RSM) Optimization of Extraction Conditions

#### 2.3.1. Model Analysis

Table 2 displays the quadratic response surface regression equation and derivative equations of the lignanoid extraction rate in relation to the actual value of each factor following the fitting of the regression. The model of the lignanoid extraction rate has a value of *p* < 0.0001 (Table S2), suggesting that the regression equation has a high degree of dependability and the result is meaningful. The lack-of-fit item *p* > 0.05 means that it is not significant in comparison to the pure error; it also proves that the real value is quite consistent with the expected value and the model has a high degree of fitting.

**Figure 3:**
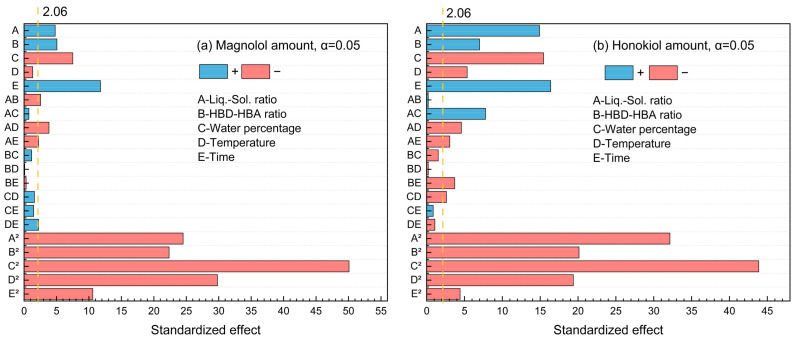
The standardized Pareto chart of the main effects for (**a**) magnolol amount and (**b**) honokiol amount.

The standardized Pareto chart (Figure 3) was used to illustrate the effects of factors on the magnolol and honokiol amount. It was found that the *t*-test results of magnolol and honokiol were both 2.06 at the 95% confidence level. There are two categories of effects: positive and negative. Figure 3a shows that the magnolol amount is positively impacted by A, B, E, AB, AC, and CE, while other factors have negative impacts. For the honokiol amount, the factors A, B, E, AC, BC, BD, CD, CE, and DE displayed positive effects (Figure 3b). Furthermore, water percentage × water percentage (C^2^) has the highest influence for the amount of both magnolol and honokiol since it stretches the furthest.

**Figure 4:**
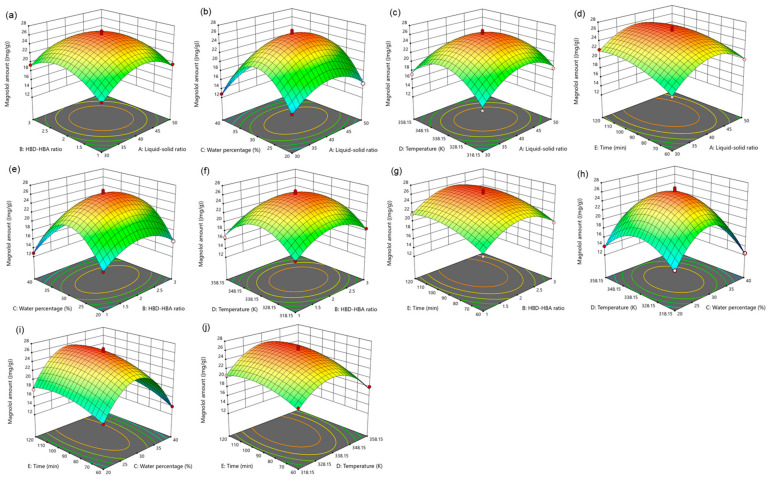
The response surface of the effect of independent variable interactions on magnolol amount (**a**): liquid–solid ratio and HBD-HBA ratio; (**b**): liquid–solid ratio and water percentage; (**c**): liquid–solid ratio and temperature; (**d**): liquid–solid ratio and time; (**e**): HBD-HBA ratio and water percentage; (**f**): HBD-HBA ratio and temperature; (**g**): HBD-HBA ratio and time; (**h**): water percentage and temperature; (**i**): water percentage and time; (**j**): temperature and time.

**Figure 5:**
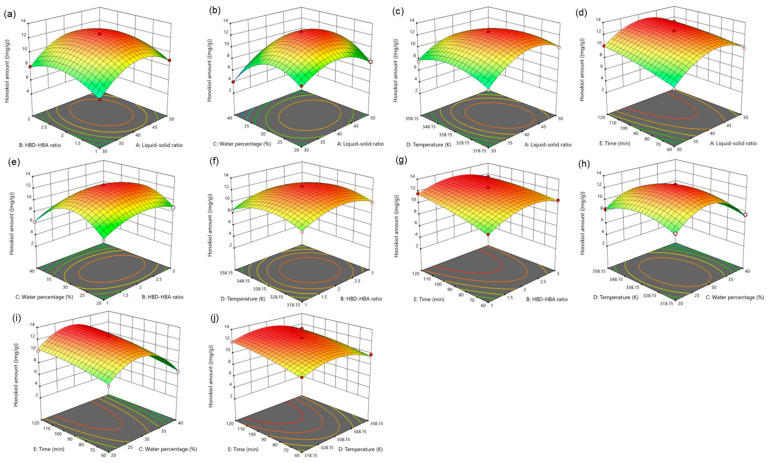
The response surface of the effect of independent variable interactions on honokiol amount (**a**): liquid–solid ratio and HBD-HBA ratio; (**b**): liquid–solid ratio and water percentage; (**c**): liquid–solid ratio and temperature; (**d**): liquid–solid ratio and time; (**e**): HBD-HBA ratio and water percentage; (**f**): HBD-HBA ratio and temperature; (**g**): HBD-HBA ratio and time; (**h**): water percentage and temperature; (**i**): water percentage and time; (**j**): temperature and time.

For five influencing factors, the effects on the amount of magnolol are arranged in the following order: E (time) > C (water percentage) > B (HBD-HBA ratio) > A (liquid–solid ratio) > D (temperature). The influence on the amount of honokiol is arranged in the following order: E (time) > C (water percentage) > A (liquid–solid ratio) > B (HBD-HBA ratio) > D (temperature). In addition, the link between the impact variables is clearly shown by the response surface map (Figure 4 and Figure 5).

#### 2.3.2. Determination and Verification of Optimal Condition

Based on the optimal parameters (liquid–solid ratio of 40.65 mL/g, HBD-HBA ratio of 2.06, water percentage of 29.3%, temperature of 337.59 K, and time of 106.84 min) through RSM optimization, the maximum lignanoid (magnolol and honokiol) amount of 39.21 mg/g was obtained. The verification test was conducted to evaluate the dependability of the optimization results, and the highest lignanoid amount was 39.18 mg/g. Moreover, it can be seen that the prediction model is effective and the experimental optimization parameters are reliable, as indicated by the relative standard deviation (RSD%) of 0.25% (Supplementary Table S2).

### 2.4. Extraction Kinetics

**Figure 6:**
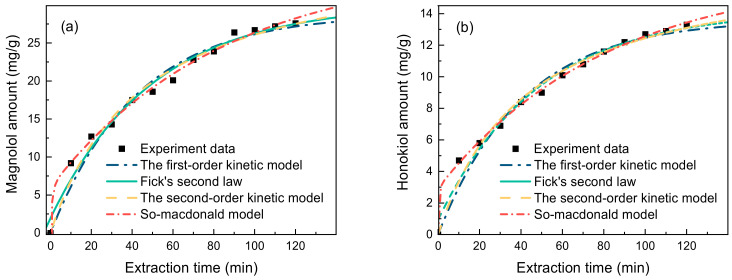
Kinetic fitted curves of (**a**) magnolol amount and (**b**) honokiol amount (liquid–solid ratio of 40 mL/g, HBD-HBA ratio of 2, water percentage of 30%, extract temperature of 338.15 K, and time of 90 min).

For the current extraction method to be applied industrially, extraction kinetic experiments need to be performed, the kinetic equations need to be established, and the mass transfers need to be explored. Thus, in order to fit the extraction kinetics of lignanoids, four primary kinetic models were used: the first-order kinetic model, Fick’s second law kinetic model, the second-order kinetic model, and the So–Macdonald model. With a higher correlation coefficient (R^2^) and a lower residual sum of squares (RSS), the So–Macdonald model outperformed the other three kinetic models in matching experimental data, as shown in Figure 6 and Table 3.

### 2.5. The Biological Activity

#### 2.5.1. The Antioxidant Activity

**Figure 7:**
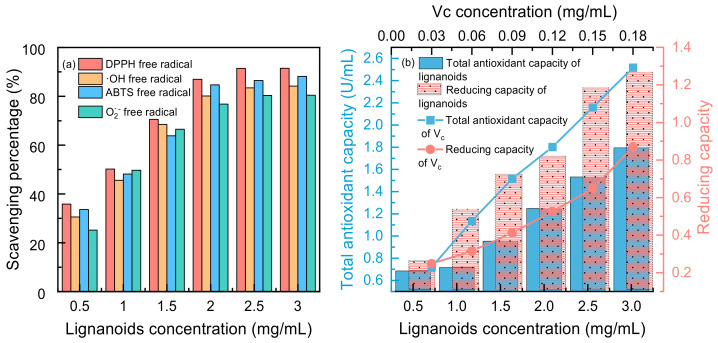
(**a**) Scavenging percentage and (**b**) total antioxidant and reducing capacity of lignanoid extract under different concentrations.

Four free radicals were used to test the antioxidant activity of the lignanoid extract, and the findings are displayed in Figure 7. It was demonstrated that lignanoids have potent antioxidant effects and can effectively scavenge four types of free radicals: DPPH free radicals, ·OH free radicals, ABTS free radicals, and O_2_^−^ free radicals. Moreover, Figure 7b illustrates that the total antioxidant capacity and reducing capacity of the lignanoid extract had a dose-dependent impact within a specific concentration range of 0.5–3 mg/mL. At a concentration of 3 mg/mL, the total antioxidant capacity of the lignanoid extract was equal to 0.12 mg/mL of the V_c_ solution. Therefore, the lignanoid extract has a good total antioxidant and reducing capability within a specific concentration range, as demonstrated by the preceding data.

#### 2.5.2. The Antibacterial Activity

**Figure 8:**
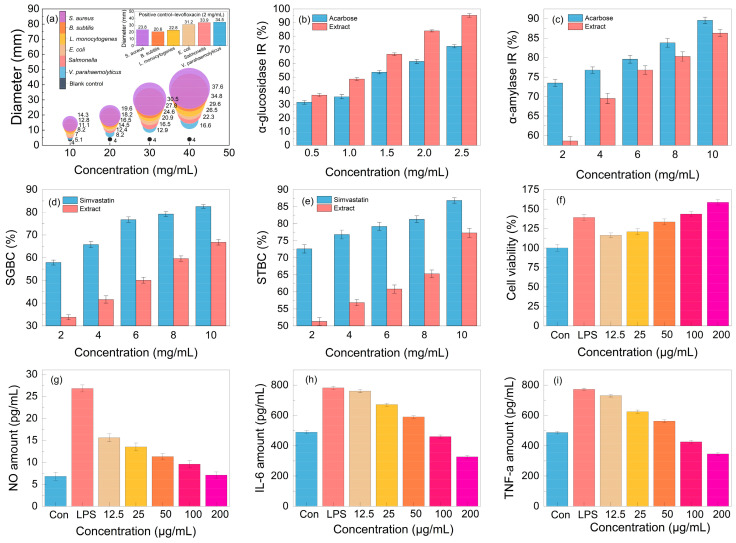
The (**a**) antibacterial activity, (**b**) α-glucosidase inhibition rate (IR), (**c**) α-amylase inhibition rate (IR), (**d**) sodium glycinate binding capacity (SGBC), (**e**) sodium taurocholate binding capacity (STBC), (**f**) cell viability, and (**g**–**i**) anti-inflammatory effect of the lignanoid extract.

Aiming to test the antibacterial activity of the lignanoid extract, six pathogens were employed in the tests. As seen in Figure 8, the lignanoid extract exhibited significant inhibitory effects on all evaluated Gram-negative and -positive bacteria. Compared to Gram-negative bacteria, the lignanoid extract exerted a greater inhibitory effect on positive bacteria. One explanation might be because Gram-positive bacteria possess a larger negative surface charge than Gram-negative bacteria and a higher concentration of peptidoglycan in their cell walls rather than lipopolysaccharide, which resulted in a diminished ability to defend cells [44]. In addition, the inhibitory effect of the lignanoid extract on certain bacteria is higher than that of the positive control group, levofloxacin (2 mg/mL). At a lignanoid concentration of 40 mg/mL, the antibacterial effect of the extract was as follows: *Staphylococcus aureus* (37.6 ± 1.46 mm) > *Bacillus subtilis* (34.8 ± 1.49 mm) > *Listeria monocytogenes* (29.6 ± 1.42 mm) > *Escherichia coli* (26.5 ± 1.43 mm) > *Salmonella* (22.3 ± 1.41 mm) > *Vibrio parahaemolyticus* (16.6 ± 1.45 mm).

#### 2.5.3. The In Vitro Hypoglycemic and Hypolipidemic Activity

The in vitro hypoglycemic effect of the lignanoid extract was evaluated based on the α-glucosidase inhibition rate (IR) and α-amylase inhibition rate (IR), which are the two primary enzymes for the metabolism of glucose [45]. As can be shown in Figure 8b, the lignanoid extract significantly outperformed the positive control acarbose in its ability to inhibit α-glucosidase. In particular, the α-glucosidase inhibition rate reached 95.3% at the lignanoid concentration of 2.5 mg/mL. The result of the α-amylase inhibition rate is depicted in Figure 8c; it can be seen that the α-amylase inhibition rate increased with the increase in the lignanoid concentration, but is lower than the acarbose positive control. Consequently, the in vitro hypoglycemic effect of the lignanoid extract was significant.

The cholate binding technique was employed to assess the in vitro hypolipidemic effect of the lignanoid extract. Theoretically, if the drug was mixed with bile salt, the concentration of bile salt in the body could decrease to maintain the acid balance of bile, promote the breakdown of fat in the liver, and eventually assist in lowering blood lipid levels [46]. The two main important bile salts for human bile synthesis and lipolysis are sodium taurocholate and sodium glycocholate. Figure 8d,e illustrate a significant dose–effect relationship whereby the capacity of the lignanoid extract to adsorb taurocholate and glycocholate increased with concentration. Therefore, the in vitro hypolipidemic effects of the lignanoid extract were significant.

#### 2.5.4. The Immunomodulatory and Anti-Inflammatory Activity

An integral part of the innate immune system are macrophages. These cells are in charge of most immunological processes, including host inflammation. Hence, the effect on RAW264.7 cell activity and proliferation of the lignanoid extract was investigated. As seen in Figure 8f, the lignanoid extract could enhance RAW264.7 cell proliferation within a specific concentration range. When compared to uninfected controls, the lignanoid extract significantly boosted RAW264.7 cell proliferation to the same level as lipopolysaccharide (LPS) at a dose of 200 μg/mL.

To assess the anti-inflammatory effects of the lignanoid extract, we evaluated the inhibitory capability of NO, IL-6, and TNF-α in LPS-stimulated RAW264.7 cells. It was found that LPS stimulation significantly increased the production of NO, IL-6, and TNF-α in macrophages, as shown in Figure 8g–i. Subsequent to the lignanoid extract culture, macrophages released significantly less NO, IL-6, and TNF-α, indicating that the lignanoid extract may have an inhibitory effect on macrophage inflammatory factors.

### 2.6. Correlation Analysis

**Figure 9:**
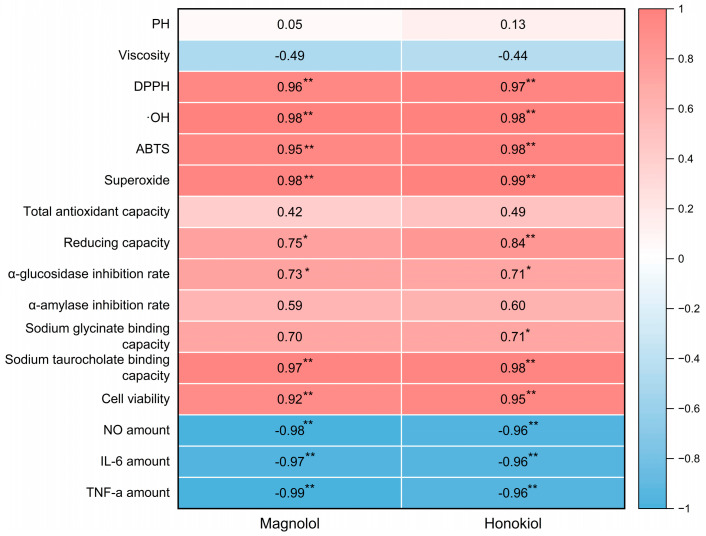
The heatmap of magnolol and honokiol with the physicochemical properties of DESs and the biological activity of the lignanoid extract. * and ** denote a significant association at the *p* < 0.05 and *p* < 0.01 levels, respectively.

The Pearson correlation coefficients were calculated in order to investigate the relationship between active components, the biological activity of the lignanoid extract, and the physicochemical properties of DESs. The findings are shown as a heatmap (Figure 9), which reveals a high connection between biological activity and active components. Positive correlations were observed between active components (magnolol and honokiol) and the DPPH (*p* < 0.01), ·OH (*p* < 0.01), ABTS (*p* < 0.01), superoxide (*p* < 0.01), sodium taurocholate binding capacity (*p* < 0.01), and cell viability (*p* < 0.01), which demonstrate that the lignanoid extract had a favorable effect on radical scavenging and cell viability. On the other hand, active components (magnolol and honokiol) had a negative correlation (*p* < 0.01) with the amounts of key inflammatory markers (NO, IL-6, and TNF-α). Therefore, the stronger anti-inflammatory effects of the lignanoid extract were indicated by greater correlation coefficient values of NO, IL-6, and TNF-α, which further support the predictive significance of the lignanoid extract for biological activity.

## 3. Material and Methods

### 3.1. Raw Materials and Chemicals

The raw materials of *Magnolia officinalis* residues (MORs) were supplied by Hunan Heguang Biotechnology Co., Ltd. (Yongzhou, China), which were crushed and sieved with a 20–40-mesh sieve (425–850 μm) and placed in a vacuum dryer for subsequent experiments. Standard samples of magnolol and honokiol were provided by China National Pharmaceutical Group Chemical Reagent Co., Ltd. (Shanghai, China), and other reagents were utilized without additional purification after being purchased from Fuchen Chemical Reagent Co., Ltd. (Tianjin, China). Fresh deionized water was utilized throughout the whole experimental procedure.

### 3.2. Preparation of Deep Eutectic Solvents

**Figure 10:**
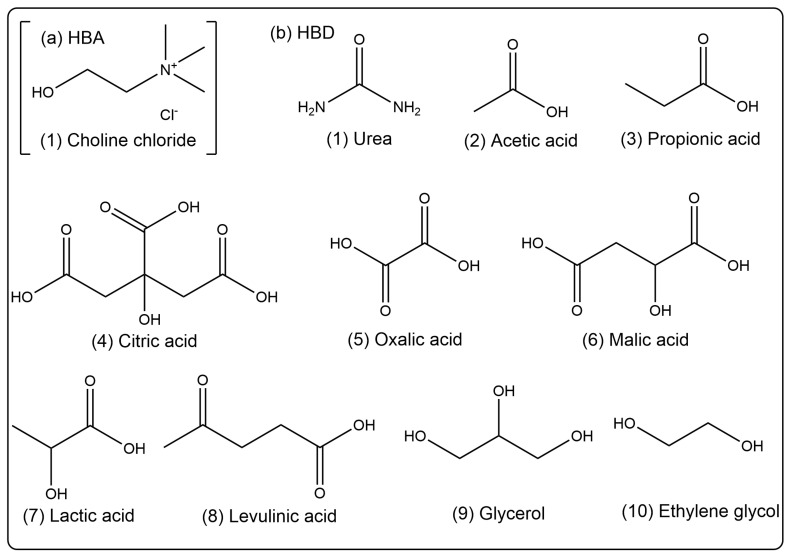
Chemical structures of (**a**) HBA (Choline chloride) and (**b**) HBDs (urea, acetic acid, propionic acid, citric acid, oxalic acid, malic acid, lactic acid, levulinic acid, glycerol, and ethylene glycol).

We used the previous preparation method of the research group [47]. Different kinds of HBAs (Choline chloride, ChCl) and HBDs (urea, acetic acid, propionic acid, citric acid, oxalic acid, malic acid, lactic acid, levulinic acid, glycerol, and ethylene glycol) were mixed and stirred with a magnetic stirrer for 30–60 min at 353 K to form a clear liquid. Then, 20% (*v*/*v*) of water was added and stirred for 30 min obtain homogeneous and clear DESs (ChUre, ChAce, ChPro, ChCit, ChOxa, ChMal, ChLac, ChLev, ChGly and ChEG). The specific chemical structures of HBA and HBD are depicted in Figure 10.

### 3.3. Characterization of MORs before and after Extraction

The pore volumes of samples were measured at 77.3 K using the Brunauer–Emmett–Teller method. Prior to measurement, samples were degassed for 12 h at 373.15 K.

### 3.4. Selection of Optimal DES for Lignanoid Extraction

Firstly, a 10 g MOR was added into a flask, and the DES was subsequently added under similar conditions (DES composition molar ratio of 1:1, liquid–solid ratio of 30 mL/g, water percentage of 30%, extraction temperature of 318.15 K, and time of 60 min). After the reaction, the reactant was filtered, and the residues were rinsed with water and dried to constant mass. After that, a 0.22 µm microporous membrane (Jinteng Co., Ltd., Tianjin, China) was employed to filter the extracted solution. Lastly, the content of each lignanoid component in the extract was determined using high-performance liquid chromatography (HPLC).

### 3.5. Selection of Optimal DESs: Single-Factor Experiment Design

After weighing and transferring 10 g of the MOR into a flask, the DES was added. The effects of the DES composition molar ratio (1:1, 1:2, 1:3, and 1:4), liquid–solid ratio (10, 20, 30, 40, 50 and 60 mL/g), water percentage (0, 10, 20, 30, 40 and 50%), extraction temperature (298.15, 318.15, 338.15, 358.15 and 378.15 K), and time (30, 60, 90 and 120 min) on the extraction rate of total lignanoids were determined. The data were obtained by repeating each of the treatment groups 3 times.

### 3.6. HPLC Measurement and Analysis

The HPLC measurement and analysis were performed on a Shimadzu LC-20A High-performance liquid chromatograph with an SPD-20A UV detector and an Amethyst C18-H column. The mobile phase used for liquid chromatography was methanol/water/acetonitrile = 45:35:20 (*V*/*V*/*V*). The conditions were a flow rate of 1.0 mL/min, a detection wavelength of 294 nm, a column temperature of 298.15 K, and an injection volume of 10 μL. To guarantee accuracy, three duplicates of each sample were completed. The HPLC chromatograms of the standard compounds and lignanoid extract are depicted in Figure S1.

### 3.7. Response Surface Optimization Design of Experiments

Based on the single-factor test results, ChLev was employed as the best DES for enhancing the extraction of lignanoids. Five variables—the DES composition molar ratio, liquid–solid ratio (mL/g), water percentage (%), extraction temperature (K), and time (min)—were chosen as the experimental control factors, and the lignanoid amount was employed as the response result. Table 4 displays the factor level coding table for the Box–Behnken experimental design, and Supplementary Table S1 displays the specific factor level design.

### 3.8. Extraction Kinetic Models

To make better sense of the experimental results, four main kinetic models were employed: the first-order kinetic model, Fick’s second law kinetic model, the second-order kinetic model, and the So–Macdonald model. The specific calculation equations are depicted in the Supplementary Materials. Furthermore, the correlation degree between experimental and predicted values was verified.

### 3.9. Data Analysis

In order to evaluate the model reliability, the correlation coefficient squared (R^2^), residual sum of squares (RSS), and chi-square (χ^2^) were calculated and analyzed by OriginPro 2021 software.

### 3.10. The Biological Activity Test

The specific antioxidant and antibacterial activity testing processes are depicted in the Supplementary Materials.

## 4. Conclusions

Ten types of green, environmentally friendly, and low-cost DESs were synthesized for the extraction of lignanoids (magnolol and honokiol) from *Magnolia officinalis* residual waste biomass. ChLev exhibited the greatest extraction efficiency among them. The highest lignanoid amount of 39.18 mg/g was achieved under the optimized parameters (liquid–solid ratio of 40.50 mL/g, HBD-HBA ratio of 2.06, water percentage of 29.3%, extraction temperature of 337.65 K, and time of 107 min). Furthermore, this work confirmed that the So–Macdonald model can effectively describe and comprehend the kinetic extraction process of lignanoids. In addition, the lignanoid extract exhibited excellent antioxidant capacity and the ability to scavenge four free radicals (DPPH free radical, ·OH free radical, ABTS free radical, and O_2_^−^ free radical). The investigation of antibacterial activity discovered that the lignanoid extract exerted high antibacterial ability against all tested pathogens in the order of *Staphylococcus aureus* (37.6 ± 1.46 mm) > *Bacillus subtilis* (34.8 ± 1.49 mm) > *Listeria monocytogenes* (29.6 ± 1.42 mm) > *Escherichia coli* (26.5 ± 1.43 mm) > *Salmonella* (22.3 ± 1.41 mm) > *Vibrio parahaemolyticus* (16.6 ± 1.45 mm) with a 40 mg/mL concentration of lignanoids. Additionally, significant hypoglycemic and hypolipidemic activity was also demonstrated by the lignanoid extract. Meanwhile, the lignanoid extract had significant anti-inflammatory effects, as demonstrated by the immunomodulatory activity test, and it was also able to increase macrophage proliferation and regulate immunological activity. We believe that our work may open up new avenues for the eventual development of an innovative, environmentally friendly, and highly effective method for the specific extraction and application of natural products. In a follow-up work, the concentration of biomass in DESs can be further increased to improve the economic feasibility on an industrial scale.

## Figures and Tables

**Table 1:** Extraction effect of various DESs and textural characteristics of the fresh and extracted samples *.

DES	PH	Viscosity (mPa·s)	Amount (mg/g) *		S_BET_ (m^2^/g)		V_pore_ (cm^3^/g)	
			Magnolol	Honokiol	Fresh	Extracted	Fresh	Extracted
ChUre	7.26	16.5	-	1.68 ± 0.38	1.65	3.26	0.09	0.11
ChAce	2.85	15.6	14.58 ± 1.15	6.35 ± 0.45	1.65	14.25	0.09	0.18
ChPro	2.26	13.8	16.89 ± 1.06	7.68 ± 0.35	1.65	16.35	0.09	0.19
ChCit	0.48	27.6	5.68 ± 1.21	3.56 ± 0.26	1.65	7.52	0.09	0.14
ChOxa	0.11	50.8	3.56 ± 1.35	2.56 ± 0.32	1.65	4.65	0.09	0.12
ChMal	0.22	560.1	2.58 ± 1.12	1.35 ± 0.15	1.65	3.28	0.09	0.11
ChLac	0.85	26.9	9.58 ± 1.08	4.68 ± 0.38	1.65	10.86	0.09	0.16
ChLev	1.09	103.0	18.79 ± 1.23	9.56 ± 0.32	1.65	19.65	0.09	0.21
ChGly	6.75	876.2	4.98 ± 1.32	3.18 ± 0.49	1.65	6.86	0.09	0.13
ChEG	6.89	378.1	9.68 ± 1.05	5.92 ± 0.45	1.65	13.54	0.09	0.17

Extraction conditions: DES composition molar ratio of 1:1, liquid–solid ratio of 30 mL/g, water percentage of 30%, temperature of 318.15 K, and extraction time of 60 min. * The lignanoid (honokiol and magnolol) amount (mg/g) is calculated as mg lignanoids/g raw material.

**Table 2:** Model equations and derivative equations with coded factors for magnolol amount and honokiol amount.

Model	Equation
Magnolol amount (Ma)	Magnolol amount (mg/g) = 26.02 + 0.5625A + 0.5938B − 0.8812C − 0.1563D + 1.38E − 0.6000AB + 0.1750AC − 0.9000AD − 0.5250AE + 0.2750BC + 0.0250BD − 0.0750BE + 0.3750CD + 0.3500CE + 0.5250DE − 3.89A² − 3.55B² − 7.95C² − 4.73D² − 1.68E²
Honokiol amount (Ha)	Honokiol amount (mg/g) = 12.42 + 0.8525A + 0.4000B − 0.8837C − 0.3063D + 0.9375E + 0.0250AB + 0.8900AC − 0.5250AD − 0.3500AE − 0.1750BC − 0.0250BD − 0.4250BE − 0.3000CD + 0.1000CE − 0.1250DE − 2.49A² − 1.56B² − 3.40C² − 1.50D² − 0.3425E²
αMa/αA	αMa/αA = 0.5625 − 0.6000AB + 0.1750C − 0.9000D − 0.5250E − 7.78A
αMa/αB	αMa/αB = 0.5938 − 0.6000B + 0.2750B + 0.0250B − 0.0750B − 7.10B
αMa/αC	αMa/αC = −0.8812 + 0.1750A + 0.2750B + 0.3750D + 0.3500E − 15.90C
αMa/αD	αMa/αD = −0.1563 − 0.9000A + 0.0250B + 0.3750C + 0.5250E − 9.46D
αMa/αE	αMa/αE = 1.38 − 0.5250A − 0.0750B + 0.3500C + 0.5250E − 3.36E
αHa/αA	αHa/αA = 0.8525 + 0.0250B + 0.8900C − 0.5250D − 0.3500E − 4.98A
αHa/αB	αHa/αB = 0.4000 + 0.0250A − 0.1750C − 0.0250D − 0.4250E − 3.12B
αHa/αC	αHa/αC = −0.8837 + 0.8900A − 0.1750B − 0.3000D + 0.1000E − 6.80C
αHa/αD	αHa/αD = −0.3063 − 0.5250A − 0.0250B − 0.3000C − 0.1250E − 3.00D
αHa/αE	αHa/αE = 0.9375 − 0.3500A − 0.4250B + 0.1000C − 0.1250E − 0.685E

**Table 3:** Modeling results of experimental data.

Model	Magnolol Amount			Honokiol Amount		
	R^2^	RSS	Chi-Square	R^2^	RSS	Chi-Square
First-order kinetic model	0.9783	20.7242	0.9554	0.9704	4.2993	0.4649
Fick’s second law	0.9806	15.8216	2.4313	0.9827	3.1655	1.1706
Second-order kinetic model	0.9847	13.3588	0.551	0.9869	2.6110	0.2684
So–Macdonald model	0.9956	3.6209	0.0857	0.9985	0.2669	0.0165

**Table 4:** Design factor levels and codes for lignanoid extraction.

Name	Units	Type	Low	High
Liquid–solid ratio	mL/g	Factor	30	50
HBD-HBA ratio		Factor	1	3
Water percentage	%	Factor	20	40
Temperature	K	Factor	318.15	358.15
Time	min	Factor	60	120
Honokiol amount	(mg/g)	Response		
Magnolol amount	(mg/g)	Response		
Lignanoid amount	(mg/g)	Response		

## Data Availability

Data are contained within the article and Supplementary Materials.

## Supplementary Materials

The following supporting information can be downloaded at https://www.mdpi.com/article/10.3390/molecules29102352/s1: Figure S1: The HPLC chromatograms of standard components and lignanoid extract; Table S1: The specific factor level design for lignanoid extraction; Table S2: ANOVA and fit statistics of magnolol amount and honokiol amount. Table S3: Repeatability of lignanoid extraction.
