# Supplementary material for: Extraction and Biological Activity of Lignanoids from Magnolia officinalis Rehder & E.H.Wilson Residual Waste Biomass Using Deep Eutectic Solvents
Source: Molecules. 2024 May 16;29(10):2352. doi: 10.3390/molecules29102352 (PMC11124428; doi:10.3390/molecules29102352)
Supplement: Supplementary file 1 [file molecules-29-02352-s001.zip › molecules-2985580-supplementary.pdf]

Supplementary Materials

1. The HPLC chromatograms of standard components and lignanoids extract

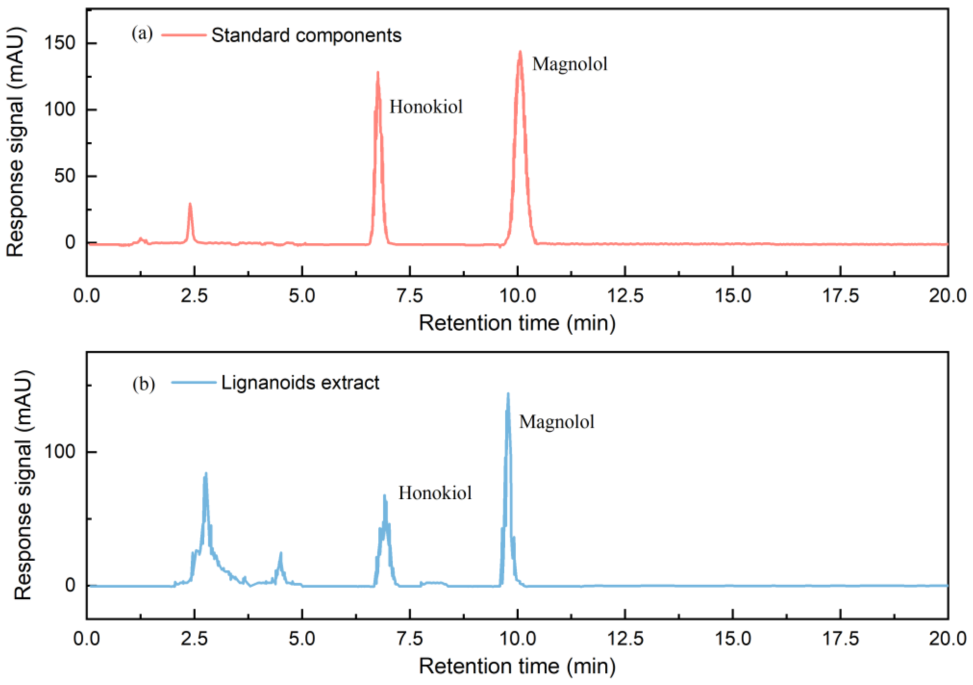

Figure S1. The HPLC chromatograms of (a) standard components and (b) lignanoids extract*. *The extract was extracted by the ChLev solvent. Detection concentration: 1.0 mg/mL (standard components) and 10 mg/mL (lignanoids extract). Detection wavelength of 294 nm.

2. Response surface methodology (RSM)

Table S1. The specific factor level design for lignanoids extraction.

Std	Run	A: Liquid–solid ratio (mL/g)	B: HBD–HBA ratio	C: Water percentage (%)	D: Extract temperature (K)	E: Extract Time (min)	Magnolol amount (mg/g)	Honokiol amount (mg/g)	Lignanoids amount (mg/g)
25	1	30	2	30	318.15	90	15.4	7.2	22.6
28	2	50	2	30	358.15	90	16.8	8.2	25.0
20	3	40	2	30	358.15	120	21.5	11.2	32.7
19	4	40	2	30	318.15	120	20.6	11.9	32.5
46	5	40	2	30	338.15	90	26.2	12.6	38.8
1	6	30	1	30	338.15	90	17.2	7.2	24.4
4	7	50	3	30	338.15	90	19.4	9.8	29.2
27	8	30	2	30	358.15	90	17.2	7.5	24.7

23	9	40	1	40	338.15	90	12.8	6.3	19.1
33	10	30	2	30	338.15	60	17.8	7.2	25.0
22	11	40	3	20	338.15	90	15.6	8.8	24.4
6	12	40	2	40	318.15	90	12.4	7.5	19.9
5	13	40	2	20	318.15	90	15.1	8.6	23.7
42	14	40	2	30	338.15	90	25.5	12.5	38.0
38	15	40	3	30	318.15	90	18.6	9.9	28.5
39	16	40	1	30	358.15	90	16.6	8.6	25.2
21	17	40	1	20	338.15	90	15.1	7.6	22.7
45	18	40	2	30	338.15	90	26.5	12.5	39.0
2	19	50	1	30	338.15	90	19.6	8.9	28.5
9	20	40	1	30	338.15	60	18.2	8.9	27.1
36	21	50	2	30	338.15	120	22.2	11.2	33.4
41	22	40	2	30	338.15	90	26.9	12.3	39.2
8	23	40	2	40	358.15	90	12.8	6.2	19.0
7	24	40	2	20	358.15	90	14.0	8.5	22.5
14	25	50	2	20	338.15	90	15.2	7.54	22.74
44	26	40	2	30	338.15	90	25.7	12.3	38.0
35	27	30	2	30	338.15	120	22.1	10.1	32.2
40	28	40	3	30	358.15	90	17.9	9.3	27.2
3	29	30	3	30	338.15	90	19.4	8.0	27.4
34	30	50	2	30	338.15	60	20.0	9.7	29.7
12	31	40	3	30	338.15	120	22.9	11.5	34.4
17	32	40	2	30	318.15	60	19.3	10.0	29.3
18	33	40	2	30	358.15	60	18.1	9.8	27.9
32	34	40	2	40	338.15	120	16.8	8.6	25.4
10	35	40	3	30	338.15	60	19.8	10.5	30.3
11	36	40	1	30	338.15	120	21.6	11.6	33.2
13	37	30	2	20	338.15	90	14.8	7.8	22.6
37	38	40	1	30	318.15	90	17.4	9.1	26.5
26	39	50	2	30	318.15	90	18.6	10.0	28.6
24	40	40	3	40	338.15	90	14.4	6.8	21.2
31	41	40	2	20	338.15	120	17.8	10.1	27.9
15	42	30	2	40	338.15	90	12.8	4.0	16.8
16	43	50	2	40	338.15	90	13.9	7.3	21.2
43	44	40	2	30	338.15	90	25.3	12.3	37.6

29	45	40	2	20	338.15	60	16.3	8.5	24.8
30	46	40	2	40	338.15	60	13.9	6.6	20.5

Table S2. ANOVA and fit statistics of magnolol amount and honokiol amount.

**(a) Magnolol amount Source**	**Sum of sq.**	**df**	**M. sq.**	***F*-Value**	***P*-value**	**Prob>F**	**Index mark**	**Value**
Model	711.89	20	35.59	161.90	< 0.0001	significant	Std. Dev.	0.4689
A-Liq.–Sol. ratio	5.06	1	5.06	23.03	< 0.0001		Mean	18.43
B- HBD–HBA ratio	5.64	1	5.64	25.66	< 0.0001		C.V. %	2.54
C-Water percentage	12.43	1	12.43	56.52	< 0.0001		R^2	0.9923
D-Temperature	0.3906	1	0.3906	1.78	0.1946		Adj. R^2	0.9862
E-Time	30.53	1	30.53	138.85	< 0.0001		Pred. R^2	0.9762
AB	1.44	1	1.44	6.55	0.0169		A.Prec.	43.4940
AC	0.1225	1	0.1225	0.5572	0.4624			
AD	3.24	1	3.24	14.74	0.0007			
AE	1.10	1	1.10	5.01	0.0343			
BC	0.3025	1	0.3025	1.38	0.2518			
BD	0.0025	1	0.0025	0.0114	0.9159			
BE	0.0225	1	0.0225	0.1023	0.7517			
CD	0.5625	1	0.5625	2.56	0.1223			
CE	0.4900	1	0.4900	2.23	0.1480			
DE	1.10	1	1.10	5.01	0.0343			
A^2	132.03	1	132.03	600.56	< 0.0001			
B^2	109.86	1	109.86	499.69	< 0.0001			
C^2	551.30	1	551.30	2507.60	< 0.0001			
D^2	195.36	1	195.36	888.60	< 0.0001			
E^2	24.67	1	24.67	112.21	< 0.0001			
Residual	5.50	25	0.2199					
Lack of Fit	3.57	20	0.1784	0.4626	0.9005	not significant		
Pure Error	1.93	5	0.3857					
Cor Total	717.38							
**(b) Honokiol amount Source**	**Sum of sq.**	**df**	**M. sq.**	***F*-Value**	***P*-value**	**Prob>F**	**Index mark**	**Value**
Model	183.74	20	9.19	175.52	< 0.0001	significant	Std. Dev.	0.2288
A-Liq.–Sol. ratio	11.63	1	11.63	222.16	< 0.0001		Mean	9.19
B-HBD–HBA ratio	2.56	1	2.56	48.91	< 0.0001		C.V. %	2.49
C-Water percentage	12.50	1	12.50	238.74	< 0.0001		R^2	0.9929
D-Temperature	1.50	1	1.50	28.67	< 0.0001		Adj. R^2	0.9873
E-Time	14.06	1	14.06	268.67	< 0.0001		Pred. R^2	0.9729
AB	0.0025	1	0.0025	0.0478	0.8288		A.Prec.	55.0709
AC	3.17	1	3.17	60.53	< 0.0001			

AD	1.10	1	1.10	21.06	0.0001	
AE	0.4900	1	0.4900	9.36	0.0052	
BC	0.1225	1	0.1225	2.34	0.1386	
BD	0.0025	1	0.0025	0.0478	0.8288	
BE	0.7225	1	0.7225	13.80	0.0010	
CD	0.3600	1	0.3600	6.88	0.0147	
CE	0.0400	1	0.0400	0.7642	0.3903	
DE	0.0625	1	0.0625	1.19	0.2849	
A^2	54.07	1	54.07	1033.09	< 0.0001	
B^2	21.22	1	21.22	405.33	< 0.0001	
C^2	100.74	1	100.74	1924.63	< 0.0001	
D^2	19.66	1	19.66	375.57	< 0.0001	
E^2	1.02	1	1.02	19.56	0.0002	
Residual	1.31	25	0.0523			
Lack of Fit	1.22	20	0.0610	3.45	0.0868	not significant
Pure Error	0.0883	5	0.0177			
Cor Total	185.05	45				

3. Repeatability test

Table S3. Repeatability of lignanoids extraction*.

**Entry**	**Lignanoids amount (mg/g)**	**Average (mg/g)**	**RSD (%) (n=7)**
1	39.18		
2	38.92		
3	38.95		
4	39.11		
5	39.08		
6	38.96		
7	38.98	39.03	0.25

*Extraction conditions: liquid–solid ratio of 40.50 mL/g, HBD–HBA ratio of 2.06, water percentage of 29.3%, extract temperature of 337.65 K and time of 107 min.

4. The biological activity test

4.1. Extraction kinetic models

Four main kinetic models (First-order kinetic model, Fick's second law kinetic model, Second-order kinetic model, and So-Macdonald model) were employed to fit the experimental values. In addition, the degree of correlation between the predicted and experimental values was examined.

First-order kinetic model equation:

$$\ln[C_e/(C_e - C_t)] = k_{obs}\cdot t \quad (1)$$

Where k_{obs} (min^{-1}) represents the apparent rate constant; C_t (mg/mL) and C_e (mg/mL) represents the concentration of lignanoids (magnolol and honokiol) amount in solution at time t and equilibrium, respectively; and t (min) represents the extraction time.

Fick's second law kinetic model equation:

$$\ln[C_e/(C_e - C_t)] = k_{obs}\cdot t + \ln(\pi^2/8) \quad (2)$$

Second-order kinetic model:

$$C_t = C_e^2\cdot k_2\cdot t/(1 + C_e\cdot k_2\cdot t) \quad (3)$$

Where k_2 (min^{-1}) represents the second-order kinetic rate constant.

So-Macdonald model:

$$C_t = C_e^w\cdot[1 - \exp(-k_w\cdot t)] + C_e^{d1}\cdot[1 - \exp(-k_{d1}\cdot t)] + C_e^{d2}\cdot[1 - \exp(-k_{d2}\cdot t)] \quad (4)$$

Where k_w (min^{-1}), k_{d1} (min^{-1}), and k_{d2} (min^{-1}) represent the extraction rate constants in washing, fast diffusion and slow diffusion stages, respectively; and

C_e^w (mg/mL), C_e^{d1} (mg/mL), and C_e^{d2} (mg/mL) represent the hypothetical concentration of lignanoids (magnolol and honokiol) at equilibrium in washing, fast diffusion and slow diffusion stages, respectively.

5. The biological activity test

5.1. The antioxidant activity test

5.1.1. DPPH free radical scavenging activity test

3 mL samples of different volume fractions and 3 mL 0.1 mmol/L DPPH-ethanol solution was mixed evenly and place it at room temperature in the dark for 30 min, then the absorbance value of the treated samples was measured at λ = 517 nm. The different concentrations of V_c solutions were positive control. The test was repeated 3 times and take the average value, specific calculation formula is as follows:

$$\text{DPPH free radical scavenging rate /\%} = (A_0 - A_s)/A_0 \times 100\% \quad (5)$$

Where A_0 represent the absorbance of blank sample, A_s represent the absorbance of sample.

5.1.2. ·OH free radical scavenging activity test

100 μL samples with different volume fractions were test according to the method in the ·OH scavenging ability test kit, The V_c solutions with different concentrations were used as the control group, and the average value was taken by 3 repeated tests.

5.1.3. ABTS free radical scavenging activity test

5 mL 7 mmol/L ABTS aqueous solution and 88 μL 140 mmol/L potassium persulfate aqueous solution was mixed evenly, and were place in dark conditions for 24 h, then were diluted by absolute ethanol to keep the absorbance of 7±0.02 at λ=734 nm. 6 mL ABTS solution and 1 mL sample solution were added it respectively, mixed evenly and stand for 6 min, Finally, the absorbance of solution was measured at λ = 734 nm. Three parallel groups were made for each group to calculate the ABTS free radical scavenging rate.

$$\text{ABTS free radical scavenging rate /\%} = (A_0 - A_s)/A_0 \times 100\% \quad (6)$$

Where A_0 represent the absorbance of blank sample, A_s represent the absorbance of sample.

5.1.4. Superoxide anion free radical scavenging activity test

3 mL 50 mmol/L Tris-HCl buffer solution with pH of 8.2 and 1.0 mL lignanoids solutions with different concentrations were mixed, and were soak in a constant temperature water bath at 298.15 K for 20 min, then 0.4 mL 25 mmol/L pyrogallol solution (pre-warmed to 298.15 K) were added and mixed evenly. After 4 min of reaction, 0.5 mL HCl solution were added to terminate the reaction, and the absorbance was measured at λ = 325 nm. The deionized water was used as the control group. The test was repeated 3 times and take the average value, specific calculation formula is as follows:

$$\text{Superoxide anion free radical scavenging rate /\%} = (A_0 - A_s)/A_0 \times 100\% \quad (7)$$

where A_0 represent the absorbance of blank sample, A_s represent the absorbance of sample.

5.1.5. Total antioxidant capacity test

6 μL samples with different volume fractions were measured according to the method by the total antioxidant capacity detection kit. The V_c solutions with different concentrations were used as controls.

5.1.6. Reducing capacity test

1 mL samples of different concentrations, 1.0 mL PBS solution (0.2 mol/L, pH=6.6) and 1.0 mL 1% $K_3Fe(CN)_6$ solution were mixed evenly, and reacted in a water bath at 333.15 K for 20 min. After cooling, 1.0 mL 10% trichloroacetic acid was added to terminate the reaction, and then was centrifuged for 10 min. 2.5 mL upper layer solution were mixed with 2.5 mL distilled water and 0.5 mL of $FeCl_3$ (0.1% w/v). Finally, the mixed solution was placed at room temperature, reacted for 10 min and the absorbance was measured at 700 nm. The V_c solutions with different concentrations were used as the control.

5.2. The antibacterial activity test

The antibacterial activity test of lignanoids extract against 6 pathogens (*Staphylococcus aureus, Escherichia coli, Listeria monocytogenes, Vibrio parahaemolyticus, Bacillus subtilis* and *Salmonella* were conducted to measure the diameter of the inhibition zone in order to evaluate the antibacterial activity. To be specific, 100 μL bacterial suspension was added in Luria-Bertani (LB)

solid medium (Haibo, Qingdao, China) and spread evenly with a spreader under aseptic conditions. Then, the solid medium was punched by a hole puncher. 0.2 mL lignanoids extract with the different concentration (10, 20, 30 and 40 mg/mL) was drawing into the hole, the levofloxacin (2 mg/mL) and sterile water were used as positive control and blank control, respectively. After 24 hours of incubation at 310.15 K, and the diameter of the inhibition zone was measured 3 times and averaged.

5.3. Hypoglycemic in vitro

5.3.1. Inhibition of α-glucosidase activity

A 96-well plate was filled with a total of 100 μL of potassium phosphate buffer (pH 6.8), 20 μL of 0.2 U/mL α-glucosidase (produced with potassium phosphate buffer), and 10 μL of 1.0 mg/mL reduced glutathione [40]. Then 20 μL of various concentrations of lignanoids extract (0.5–2.5 mg/mL) were added, mixed and incubated at 310.15 K for 15 min. Then, 20 μL of 2.5 mmol/L p-nitrophenyl-α-D-glucopyranoside (PNPG) and 80 μL of 0.2 mol/L Na_2CO_3 solution were added in sequence, the absorbance value was determined by the visible spectrophotometer at 405 nm. The inhibition rate was determined employing acarbose as the reference [52].

$$\alpha\text{-glucosidase activity inhibition rate (\%)} = [1-(A_i-A_{i0})/(A_j-A_0)] \times 100\% \quad (4)$$

Where A_j represents the absorbance of enzyme, buffer, and substrate; A_0 represents the absorbance of enzyme and buffer; A_i represents the absorbance

of enzyme, sample, buffer and substrate; and A_{i0} represents the absorbance of enzyme, sample and buffer.

5.3.2 Inhibition of α-amylase activity

100 μL of 0.2 U/mL α-amylase solution (pH 6.8) were made with phosphate buffer solution, and putted into a centrifuge tube. Then, 500 μL of various concentrations of lignanoids extract (2.0–10.0 mg/mL) were added, mixed and maintained for 20 min at 310.15 K. Next, 100 μL of 10 g/L starch solution was added and stirred at 310.15 K for 10 min. Followed that, 375 μL of 3,5–dinitrosalicylic acid (DNS) reagent was added, stirred and allowed to react for 5 min in a bath of boiling water, then cooled by flowing water and centrifuged for 5 min. Finally, the value of optical density (OD) was determined at 540 nm. The inhibition rate was obtained using acarbose as the control.

$$\alpha\text{-amylase activity inhibition rate (\%)} = [1-(A_i-A_{i0})/(A_j-A_0)] \times 100\% \quad (5)$$

Where A_i represents the absorbance of sample, enzyme, starch, and DNS, A_{i0} represents the absorbance of sample, enzyme and DNS; A_j represents the absorbance of enzyme, starch, and DNS; A_0 represents the absorbance of enzyme and DNS.

5.4. Hypolipidemic in vitro

To replicate the gastric digesting process, 0.5 mL of lignanoids extract (2.0–10.0 mg/mL), and 1.5 mL of artificial gastric fluid were putted into a centrifuge

tube, and oscillated at 310.15 K for 1 h. After that, the pH of the mixture was adjusted to 6.3 using the 0.1 mol/L NaOH solution. To simulate the intestinal environment, 2.0 mL of artificial intestinal fluid was supplied for digestion at 310.15 K for 1 h. Following that, 2.0 mL of 0.3 mmol/L sodium glycocholate and sodium taurocholate were added in each sample, and then oscillated at 310.15 K for 1 h. The supernatant was utilized to determine the amounts of sodium glycocholate and sodium taurocholate by colorimetry after centrifugation. The calculation was using simvastatin as the reference.

$$\text{Cholate binding rate (\%)} = (A_i - A_0)/A_i \times 100\% \quad (8)$$

Where, A_i represents the absorbance of cholate addition; A_0 represents the absorbance of cholate residual.

5.5. Immunomodulatory activity of extract

5.5.1. Cell culture

RAW264.7 cells were cultivated in a humid atmosphere with 5% CO_2 in RPMI-1640 complete medium at 310.15 K in a box. 10% fetal bovine serum (FBS), 100 u/mL penicillin, 1 mmol/L sodium pyruvate, and 100 mg/mL streptomycin were included in this medium.

5.5.2. Cell viability

The RAW264.7 cell viability was assessed using the MTT assay. Specifically, the RAW264.7 cell suspension (1×10^5 cells/mL) was pipetted into a

96-well plate (100 μL), where it was cultivated for 24 h at 310.15 K in a humid incubator with 5% CO_2. The cells were treated with 100 μL of different concentrations (12.5, 25, 50, 100, and 200 μg/mL) of the lignanoids extract, and cultivated for an additional 24 h after discarding the supernatant. A blank control group and a positive control group (lipopolysaccharide, 1 μg/mL) were used at the same time. Then, the supernatant was aspirated. After adding 10 μL of 5 mg/mL MTT solution, the mixture was incubated for 4 h at 310.15 K. Finally, the medium was properly aspirated before each well was given 100 μL of DMSO, shaken for 10 min, and the optical density (OD) was determined at 570 nm using a microplate reader. The calculation formula is as follows.

$$\text{Cell viability\%} = A_{sample} / A_{blank} \times 100\% \quad (9)$$

5.6. Anti-inflammatory activity of lignanoids extract

To further investigate the anti-inflammatory activity of lignanoids extract. RAW264.7 cell in logarithmic growth period was counted and their cell density was adjusted. 1 mL of cell suspension was added to a 24-well cell culture plate with $2x10^5$ cell per well, and incubated for 24 h at 310.15 K with 5% CO_2. To induce macrophage polarization, 1.0 μg/mL of lipopolysaccharide (LPS) was added for 8 h, followed by 24 h of lignanoids extract solution (12.5, 25, 50, 100, and 200 μg/mL). After centrifuging, the cell supernatant was collected to detect

the amounts of nitric oxide (NO), interleukin 6 (IL-6), and tumor necrosis factor-α (TNF-α) by a NO kit and a mouse cytokine (IL-6, TNF-α) ELISA kit.
